# Commentary: Prioritizing patients for hip fracture surgery: the role of frailty and cardiac risk

**DOI:** 10.3389/fsurg.2024.1466232

**Published:** 2024-11-13

**Authors:** Patrizia Floris, Martina Manna, Valentina Spedale, Justin S. Brathwaite, Alessio Greco, Michela Passamonte, Francesco De Filippi, Paolo Mazzola

**Affiliations:** ^1^ ASST Valtellina e Alto Lario, Morbegno Hospital, Morbegno, Italy; ^2^ School of Medicine and Surgery, University of Milano-Bicocca, Monza, Italy; ^3^ Division of Diabetes and Endocrinology, University of Minnesota, Minneapolis, MN, United States; ^4^ Struttura Complessa Continuità Clinico-Assistenziale, Dipartimento di Fragilità, ASST Lecco, Lecco, Italy; ^5^ Società Italiana di Geriatria Ospedale e Territorio (SIGOT), Sezione Lombardia, Milano, Italy; ^6^ Acute Geriatrics Unit, Fondazione IRCCS San Gerardo dei Tintori, Monza, Italy; ^7^ Clinical Neurosciences Research Area, NeuroMI – Milan Center for Neuroscience, Milano, Italy

**Keywords:** hip fracture (HFr), geriatric assessment, frailty, orthogeriatric care, orthogeriatric care program

A Commentary on Prioritizing patients for hip fracture surgery: the role of frailty and cardiac risk By Forssten MP, Mohammad Ismail A, Ioannidis I, Ribeiro MAF Jr, Cao Y, Sarani B and Mohseni S (2024). Front Surg. 11:1367457. doi: 10.3389/fsurg.2024.1367457

## Introduction

We read with interest the paper by Forssten and colleagues, which emphasizes the relevance of prioritizing surgery in older adults experiencing hip fracture (HF) (1). The authors enlighten an aspect that is usually underestimated when dealing with older adults in need for surgical procedures: the presence of frailty. This syndrome, despite growing attention, is not routinely assessed outside of geriatric practice. As Forssten et al. correctly point out, the concepts of multimorbidity and frailty often tend to be misinterpreted as factors requiring pre-operative testing for medical clearance, subsequently leading to delay in surgery rather than making it a priority. Conversely, the correct identification of frailty using validated tools should perhaps prompt the anticipation of surgery rather than delaying it, in order to reduce the potential burden of a prolonged waiting time to surgery in frail subjects in terms of poor health outcomes. To date, a previous study by Turesson et al. described the impact of care process development in a large cohort of Swedish HF patients over a 19-year observation time. The authors suggest that despite the progressive aging of the population and the higher comorbidity burden observed over time, mortality rates remained almost unchanged, indirectly showing a potential (although limited) positive role of the care process implementation (2).

## Results

To support these findings, we conducted a brief analysis on data from an Italian orthogeriatrics unit, whose referral care program and treatment path have been previously described (3, 4). Briefly, inclusion criteria for the Orthogeriatrics unit were age ≥70 years, fracture of the proximal epiphysis of the femur, and at least one of the following characteristics: ≥2 comorbid conditions, polypharmacy (>3 daily medications), anticoagulation (vitamin K antagonists or DOACs), pre-existing cognitive or neuromotor impairment, suboptimal nutritional status and hydration, hemoglobin concentration <8 g/dl, heart failure (NYHA class > II), chronic kidney disease (stage 3a or higher), hypoxemia (sO2 < 90% without oxygen support), hypotension, inadequate social support. The standardized protocol of care for orthogeriatrics patients starts from the Emergency Department and warrants hospitalization within 2 h from the arrival. Eligibility criteria for admission to the Orthogeriatrics unit are verified by the orthopedic surgeon on duty, and the usual pre-operative optimization work-up after the radiological diagnosis of HF includes chest x-ray, electrocardiogram, and routine blood tests with complete blood count, renal and liver function, nutritional indices, vitamin profile, and coagulation parameters (4). Among 866 older adults collected in our database, 388 (44.8%, group 1) were operated within 24 h and 478 (55.2%, group 2) were operated after >24 h. We did not observe significant differences in terms of mean age (85.8 vs. 85.4 years), living status, place where the fracture occurred, baseline functional status, time from the Emergency Department to ward admission, blood transfusions, and day of physical therapy initiation. As for fracture types, intracapsular ones were more prevalent in group 2 than in group 1 (51.0% vs. 34.4%), while extracapsular fractures showed an opposite trend (60.1% in group 1 vs. 40.2% in group 2, *p* < 0.001). Among comorbidities at baseline, only hypertension (51.8% vs. 59.0%, *p* = 0.020) and cardiac conditions (25.8% vs. 42.5%, *p* < 0.001) resulted as statistically different between group 1 and 2, respectively. However, the mean score of CIRS-comorbidity and CIRS-severity were slightly but significantly higher in group 2 than group 1 patients. To date, a history of previous cardiac ischemia or chronic heart failure is often a reason for additional workout that may delay surgery, even in the absence of acute symptoms. Although the difference was not statistically significant, we observed a higher 1-month mortality rate among patients with hip surgery performed after >24 h (3.9% vs. 6.3%, *p* = 0.112). For each patient we calculated the OFS retrospectively and observed a similar distribution between groups, with a prevalence of scores 1, 2, and 3 in both groups. Due to the limited sample size, OFS classes 0, 4, and 5 were scarcely represented, and we did not observe death events among patients with OFS = 0. For this reason, we collapsed frailty in 3 categories: non-frail of pre-frail (OFS 0–1, *n* = 304), frail (OFS 2, *n* = 405), and severely frail (OFS ≥3, *n* = 157). A multivariable logistic regression analysis showed that being frail (OR 2.93, 95% Confidence interval: 1.01–8.58) or severely frail (OR 5.27, 95% CI: 1.65–16.85) independently predicted mortality, even after adjusting for age, sex, and time to surgery. Comorbidity, expressed by the CIRS-comorbidity score, also increased the relative risk of death with an OR 1.65 (95% CI: 1.34–2.04).

Similarly to a previous research in which we considered the impact of surgical delay and pre-existing functional disability on mortality risk (5), we divided the study population in 4 groups: (A) low frailty (OFS 0–1), surgery within 24 h (reference group); (B) frailty (OFS ≥2), surgery within 24 h; (C) low frailty, surgery delayed after 24 h; (D) frailty, surgery delayed after 24 h. Interestingly, we found that being frail (OFS ≥2) and delaying surgery after 24 h (i.e., belonging to group D) was related with the highest rate (9.0%, *n* = 28) and highest risk of death (OR 4.42, 95% CI: 1.32–14.80, *p* < 0.001).

## Discussion

Forssten and colleagues showed that surgical delay has a negative impact on survival among older adults, and that the higher the level of frailty or cardiac risk, the higher the mortality risk. Even though frailty is a multi-domain concept, if we consider one of its determinants - namely functional disability - we previously observed that patients admitted with HF and having pre-existing impairment in the activities of daily living show significantly poorer survival if their surgery is delayed more than 48 h, compared to those operated earlier (<48 h) (5). Using a similar framework to calculate the OFS in a smaller cohort, our findings are consistent with those by Forssten et al. This underlines the importance of a comprehensive evaluation when scheduling HF surgery in older adults, which could help stratify them and give surgical priority to those who will benefit most.

A recent editorial by Hernigou et al. (6) launched a critical message for the future: the world is not ready to treat an absolute number of femoral fractures that is growing rapidly. As a matter of fact, the orthopedists’ workforce is currently insufficient to face the growth of the very old and centenarian population worldwide in terms of number of expected fractures that they will experience. Therefore, addressing this challenge will require a multifaceted strategy, including a strict collaboration with healthcare professionals with geriatric competences that will support decision-making and a rational prioritization of hip fracture surgery in high-risk groups of patients.

We strongly agree with Forssten and colleagues, especially in contexts of limited resources, in promoting the assessment of frailty and to guide surgeons in reducing the waiting time for surgery accordingly. As observed worldwide, the Italian scenario confirms that avoiding delay in HF surgery for all patients seems far from the current reality, and that healthcare resources must be carefully allocated. We suggest the implementation of a comprehensive geriatric evaluation across different surgical settings, because of its ability to better characterize each patient and to help surgeons in the final decision-making about prioritizing surgery.
